# Identification of lineage‐specific *cis*–*trans* regulatory networks related to kiwifruit ripening initiation

**DOI:** 10.1111/tpj.17093

**Published:** 2024-10-27

**Authors:** Eriko Kuwada, Kouki Takeshita, Taiji Kawakatsu, Seiichi Uchida, Takashi Akagi

**Affiliations:** ^1^ Graduate School of Environmental and Life Science Okayama University Okayama 700‐8530 Japan; ^2^ Department of Advanced Information Technology Kyushu University Fukuoka 819‐0395 Japan; ^3^ Institute of Agrobiological Sciences National Agriculture and Food Research Organization Tsukuba 305‐8602 Ibaraki Japan; ^4^ Japan Science and Technology Agency PRESTO Kawaguchi 332‐0012 Saitama Japan

**Keywords:** *cis*‐regulatory elements, deep learning, ethylene, ripening

## Abstract

Previous research on the ripening process of many fruit crop varieties typically involved analyses of the conserved genetic factors among species. However, even for seemingly identical ripening processes, the associated gene expression networks often evolved independently, as reflected by the diversity in the interactions between transcription factors (TFs) and the targeted *cis*‐regulatory elements (CREs). In this study, explainable deep learning (DL) frameworks were used to predict expression patterns on the basis of CREs in promoter sequences. We initially screened potential lineage‐specific CRE–TF interactions influencing the kiwifruit ripening process, which is triggered by ethylene, similar to the corresponding processes in other climacteric fruit crops. Some novel regulatory relationships affecting ethylene‐induced fruit ripening were identified. Specifically, ABI5‐like bZIP, G2‐like, and MYB81‐like TFs were revealed as *trans*‐factors modulating the expression of representative ethylene signaling/biosynthesis‐related genes (e.g., *ACS1*, *ERT2*, and *ERF143*). Transient reporter assays and DNA affinity purification sequencing (DAP‐Seq) analyses validated these CRE–TF interactions and their regulatory relationships. A comparative analysis with co‐expression networking suggested that this DL‐based screening can identify regulatory networks independently of co‐expression patterns. Our results highlight the utility of an explainable DL approach for identifying novel CRE–TF interactions. These  imply that fruit crop species may have evolved lineage‐specific fruit ripening‐related *cis*–*trans* regulatory networks.

## Significance Statement

We applied explainable AI (or X‐AI) frameworks to screen gene networks involved in ethylene‐induced kiwifruit ripening, after which we experimentally validated the relationships and functions of these gene networks, with the underlying mechanisms possibly specific to the kiwifruit ripening process. Our results reflect the importance of the lineage‐specific evolution of regulatory networks affecting ethylene‐induced fruit ripening.

## INTRODUCTION

*Cis*‐regulatory elements (CREs), which are present in gene promoter regions and recognized by transcription factors (TFs), are important for the diversification of gene expression. They rapidly evolved in a lineage‐specific manner, through recent whole genome duplication events (Roulin et al., 2013), or during domestication events (Alonge et al., 2020). In horticultural crops, such as tomatoes, grapes, olives, and apples, lineage‐specific natural variations in CREs have often resulted in novel gene functions influencing crop quality (Alonge et al., 2020; Espley et al., 2009; Kobayashi et al., 2004; Unver et al., 2017). These variations may be the driving force behind the diversification of crop traits. However, relatively few of the contributing CREs have been identified because of their complex regulatory effects on gene expression.

Fruit ripening is an important stage that determines fruit quality‐related characteristics, including shelf‐life, flavor, firmness, and nutritional value. Fruits can be classified into two main groups, namely climacteric and non‐climacteric fruits, which are characterized by marked increases in the respiration rate and ethylene production, thereby triggering the ripening process. The common mechanism inducing the ripening of climacteric fruits involves an increase in ethylene production. However, the molecular mechanisms regulating the ripening process may differ among fruit species. In tomato, which is a representative climacteric fruit, key fruit‐ripening genes were derived from tomato‐specific *cis*‐evolution, with novel expression patterns after a lineage‐specific genome duplication event (Tomato Genome Consortium [TGC], 2012). Indeed, a key tomato‐ripening gene (*RIN*) encoding a MADS‐box TF reportedly acquired novel tomato‐specific functions (Ito et al., 2017; TGC, 2012). Although the upstream steps required for ethylene perception and signal transduction are likely similar among climacteric fruit species, there may be substantial differences in the key TFs involved in fruit ripening (Lü et al., 2018). Therefore, focusing on lineage‐specific *cis*–*trans* regulatory networks related to the fruit ripening process may clarify the evolution and molecular basis of fruit ripening.

Kiwifruit (*Actinidia chinensis* or *Actinidia deliciosa*) is a major climacteric fruit crop. Notably, the activation of the ripening process in some kiwifruit cultivars depends on exposure to exogenous ethylene. The initiation of kiwifruit ripening can be precisely controlled with exogenous ethylene treatment, which might be advantageous for the characterization of rapid changes in regulatory networks in ripening. Natural variations in ripening behavior are thought to be wide in kiwifruit, although the domesticated cultivars have narrow diversity in ripening, which is likely because kiwifruit breeding involving wild species native to China started relatively recently (i.e., in the 1900s) (Ferguson & Huang, 2007). Furthermore, unlike other climacteric fruits, ripening in kiwifruit is also triggered by low temperatures (Asiche et al., 2018; Mworia et al., 2012). These specific processes may involve a ripening mechanism different from other climacteric fruit species, making it important to study it independently of model crop insights.

Previous studies detected similarities in some of the important steps of fruit ripening‐related molecular pathways between kiwifruit and other fruit species, including steps involving ethylene perception, TFs (e.g., NAC‐like TFs; Fu et al., 2021), and cell wall degradation (Atkinson et al., 2009; Fu et al., 2021). However, transcriptome analyses revealed gene expression changes that are potentially specific to kiwifruit ripening (Asiche et al., 2018). More specifically, these expression patterns were inconsistent with the ripening process of other model fruit species, including tomato (Shinozaki et al., 2018).

**Figure 1:**
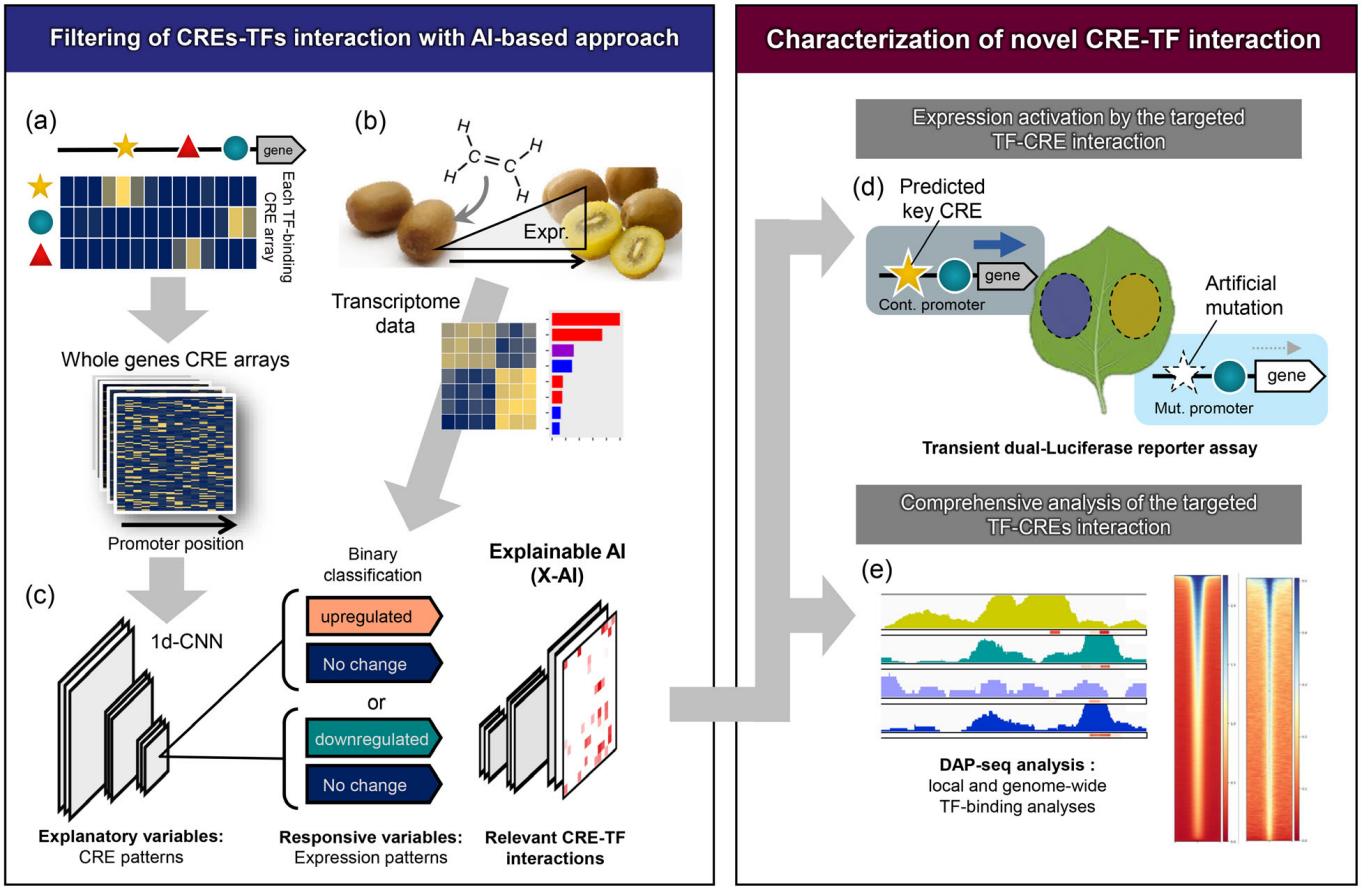
Schematic flow to identify *cis*‐regulatory elements (CREs)–transcription factors (TFs) interactions in kiwifruit ripening by integrative approach with deep learning (DL) and physiological assessment. (a) DL framework to predict CREs in a promoter (Akagi et al., 2022). This model, which was trained with the Arabidopsis cistrome datasets (O'Malley et al., 2016), was applied to the promoter sequences of the whole genes in the kiwifruit genome. (b) Transcriptome data after ethylene treatment. Genes were categorized as upregulated or downregulated. (c) One‐dimensional convolutional neural network (CNN) model was trained with the predicted whole CRE arrays to make a binary classification of expression patterns that were changed by ethylene treatment. With explainable DL techniques, the CREs and nucleotide residues relevant to the classification of expression patterns were visualized. (d, e) Experimental validation of the key *cis*–*trans* interactions predicted by our explainable DL model, for regulatory relationships of the targeted TFs and promoter regions (d), and for the direct binding ability of the TFs (e).

The objective of this study was to construct a machine learning‐based model for predicting fruit ripening‐related gene expression changes in kiwifruit according to interactions between TFs and CREs in gene promoters. Recent advances in machine learning techniques have gradually been applied for image‐based diagnoses of plant diseases (Akagi et al., 2020; Ferentinos, 2018; Ghosal et al., 2018; Masuda et al., 2021; Ramcharan et al., 2017; Singh et al., 2018; Suzuki et al., 2022) as well as for analyses of transcriptional regulatory regions (Akagi et al., 2022; Avsec et al., 2021; Mejía‐Guerra & Buckler, 2019; Meng et al., 2021; Wang et al., 2020; Washburn et al., 2019). On the basis of “explainable” deep learning (DL) (i.e., X‐AI), which predicts expression patterns via CRE–TF interactions in whole gene promoter regions (Akagi et al., 2022), we first screened novel CRE–TF interaction candidates responsible for the predicted gene expression during the kiwifruit ripening process (Figure 1a–c). The identified novel CRE–TF interaction candidates were physiologically characterized via transient reporter assays and protein–DNA binding assays (Figure 1d,e). Our approach, which integrated recent AI and physiological analyses, may be useful for revealing lineage‐specific *cis*–*trans* regulatory networks.

## RESULTS

### DL model predicted gene expression changes associated with kiwifruit ripening according to CREs in gene promoters

**Figure 2:**
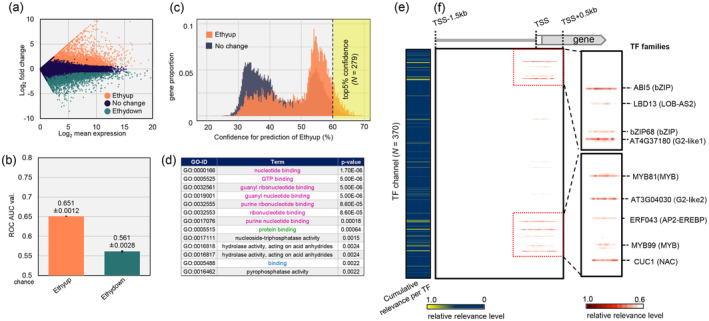
Prediction of gene expression patterns with deep learning (DL) in kiwifruit. (a) MA plot for the genes expressed in ethylene‐treated ripening kiwifruit. Ethyup, the genes significantly upregulated in the ethylene‐treated samples (false discovery rate [FDR] <0.05 with DESeq2, >1.5‐fold changes, *N* = 5576); Ethydown, the genes significantly downregulated in the ethylene‐treated samples (false discovery rate [FDR] <0.05 with DESeq2, >1.5‐fold changes, *N* = 4638); No change, the genes other than Ethyup and Ethydown. (b) Prediction performance for classification of Ethyup or Ethydown based on averaged ROC–AUC values that were calculated from 4‐fold cross validations. Bars indicated standard error (SE). (c) Distribution of the confidence for the Ethyup prediction. The confidence distributions of the actual Ethyup and control (no change) genes were statistically different (*P* < 2e^−16^, two‐sided Student's *t*‐test). The Ethyup genes with the highest 5% confidence, which will be used in subsequent analyses, are indicated in yellow. (d) Gene ontology (GO) terms significantly enriched for the genes with the highest 5% confidence for the Ethyup prediction. GO terms of general “binding,” “protein binding,” and “nucleotide binding‐related” were highlighted in blue, green, and pink, respectively. (e) Cumulative relevance levels that summarize the standardized relevance of each transcription factor (TF) over the 279 genes with the highest 5% confidence for the Ethyup prediction. (f) The sum of the positional relevance for each TF across the high‐confidence 279 genes. Nine TF channels with the highest relevance levels were annotated with the original TF names in the Arabidopsis genome TF family names are in parenthesis.

We converted the 5′ promoter regions (1.5‐kb upstream to 500‐bp downstream of the transcription start site) of the whole genes in the kiwifruit genome (*N* = 33,044; Pilkington et al., 2018) into binary CRE arrays with 370 TF channels, by using the DL model trained with Arabidopsis DAP‐seq data (O'Malley et al., 2016), according to the modified method in the previous study (Akagi et al., 2022; see Figure 1a; Figure S1, and the “Experimental procedures” section for details). For the response variables, we analyzed a transcriptomic dataset for kiwifruit ripening that was initiated by treatment with 5000 ppm exogenous propylene (an ethylene analog) (Asiche et al., 2018). We focused on binary categorized genes that were identified by DESeq as significantly upregulated or downregulated by the propylene treatment (designated as Ethyup and Ethydown, respectively) according to the following criteria: false discovery rate (FDR) <0.05 and expression level fold‐change >1.5, 3, 5, 10, and 20 (Figure 2a; Tables S1 and S2). The one‐dimensional convolutional neural network (CNN) model, which was modified to predict expression changes (Akagi et al., 2022), was trained using 370 TF‐channeled CRE arrays to classify the expression patterns into two categories: Ethyup versus Cont. or Ethydown versus Cont (Figure 1b). The models for classifying Ethyup and Cont. generated the highest receiver operating characteristic‐area under the curve (ROC‐AUC) values for >1.5‐fold changes (average ROC‐AUC = 0.651 with 4‐fold cross‐validation; Figure 2b). The accuracy of the predictions depends on the biological context. The slightly lower accuracy of this model, compared to a recent study on gene expression changes during tomato ripening (Akagi et al., 2022), is likely to be due to the differences in ripening stages. The previous study on tomato ripening focused only on very early ripening responses, in which transcriptional regulatory network (or key TF behaviors) is thought to be simpler (Akagi et al., 2022) than our case. Ethydown predictions were close to random (Figure 2b for ROC values). This tendency is consistent with the CNN prediction of down‐regulated genes in tomato ripening (Akagi et al., 2022). Similar to the tomato case, this might be hard to predict negative regulatory networks, in which transcription inhibitors would be complicatedly involved. Hence, we targeted the model for Ethyup in subsequent analyses. Although the prediction performance for Ethyup was far from perfect (ROC‐AUC = 1.0), the accuracy of a prediction depended on the model structure as well as biological contexts. In this study, we intentionally restricted the explanatory variables to the CRE array to identify new CRE–TF interactions critical for expression changes, although earlier research indicated additional parameters, including various epigenetic marks, may improve the prediction performance (Avsec et al., 2021; Singh et al., 2016).

For the classification model for Ethyup, the distribution of the prediction confidence for Ethyup genes was clearly separated from that&amp;#x000A0;for control genes (Figure&amp;#x000A0;2c). There was no significant correlation between either prediction confidence and gene expression levels (Reads Per Kilobase of exon per Million mapped reads; RPKM) or biases between the control and Ethyup genes (*P* > 0.1; Figure S2). The Ethyup genes with the highest prediction confidence (i.e., top 5%; *N* = 279; Figure 2c; Table S3) included *ethylene‐regulated nuclear protein 2* (*ERT2*) (*Acc25264.1*) and *ethylene responsive element binding factor 143* (*ERF143*) (*Acc02741.1*) as well as other genes associated with ethylene signaling. The enriched nucleotide binding‐related Gene Ontology (GO) terms among these genes included nucleotide binding (GO:0000166), GTP binding (GO:0005525), guanyl ribonucleotide binding (GO:0032561), guanyl nucleotide binding (GO:0019001), purine ribonucleotide binding (GO:0032555), ribonucleotide binding (GO:0032553), and purine nucleotide binding (GO:0017076) (Figure 2d). Additionally, the enriched GO terms protein binding (GO:0005515) and binding (GO:0005488) implied the genes contributed to general binding activities, including those involving TFs. Accordingly, although our CNN model did not specifically focus on representative ethylene‐signaling genes, it identified some of them with high confidence and was able to predict Ethyup expression changes mainly focusing on upstream transcriptional regulators (e.g., TF genes) during the kiwifruit ripening process.

### Identification of novel key CREs potentially related to kiwifruit ripening

We applied the guided backpropagation (Springenberg et al., 2015) to identify the CREs relevant to the 279 Ethyup genes predicted with the highest confidence (top 5%). The cumulative relevance of the 279 Ethyup genes was enriched in the TF channels for ABI5 (bZIP family), LBD13 (LOB‐AS2 family), AT4G37180 (G2‐like family1), bZIP68 (bZIP family), MYB81 (MYB family), AT3G04030 (G2‐like family2), ERF043 (ERF‐EREBP family), MYB99 (MYB family), and CUC1 (NAC family), which were mostly close to the transcription start site (Figure 2e). The NAC and ERF‐EREBP families include NOR‐like NAC TFs (Nieuwenhuizen et al., 2021) and ethylene response factor (ERF) TFs (Yin et al., 2010), respectively, which are critical for initiating the kiwifruit ripening process. These results suggest our DL model reflects the actual physiological reactions influencing kiwifruit ripening. Moreover, our model may be applicable for identifying CRE–TF interactions involved in activating expression.

**Figure 3:**
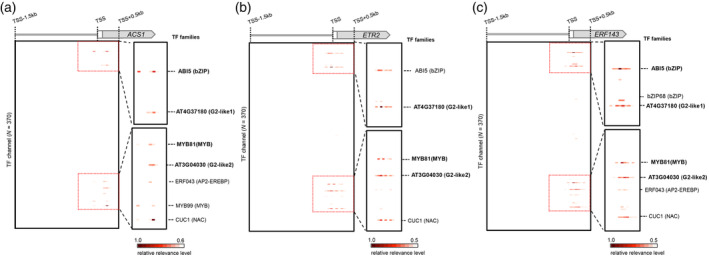
Visualization of the key *cis*‐elements for predicting expression patterns. (a–c) Identification of the *cis*‐regulatory elements (CREs) in the promoter region of *ACS1* (a), *ERT2* (b), and *ERF143* (c) that are responsible for Ethyup. Guided backpropagation on the CNN model for the Ethyup prediction identified 7, 5, and 7 transcription factor (TF) channels with high relevance levels in the *ACS1*, *ERT2*, and *ERF143* promoters, respectively. Among them, CUC1 (NAC family) and ERF043 (ERF‐EREBP family), TF families known to be associated with fruit ripening, have high cumulative relevance levels. However, there were also some TFs whose relevance to fruit ripening is little known. They were indicated in bold.

We focused on the predicted key CRE–TF interactions that activated the expression of the following three ethylene‐related genes, which were included among the Ethyup genes with high prediction confidence: *ACS1* (*Acc05955.1*), *ethylene‐regulated nuclear protein 2* (*ERT2*) (*Acc25264.1*), and *ERF143* (*Acc02741.1*). As a representative ethylene signaling/synthesis gene, *ACS* plays an important role in fruit ripening in many species, including kiwifruit (Atkinson et al., 2011; Dandekar et al., 2004; Hamilton et al., 1990; Oeller et al., 1991). *ERF143* is important for ethylene signal transduction and the regulation of ripening‐related gene transcription (Brian et al., 2021). In the *ACS1* promoter, the CREs relevant to the Ethyup prediction were enriched in the TF channels for ABI5 (bZIP family), AT4G37180 (G2‐like family1), MYB81 (MYB family), AT3G04030 (G2‐like family2), ERF043 (ERF‐EREBP family), MYB99 (MYB family), and CUC1 (NAC family) (Figure 3a). In the *ERT2* promoter, the relevant CREs were enriched in the TF channels for ABI5 (bZIP family), AT4G37180 (G2‐like family1), MYB81 (MYB family), AT3G04030 (G2‐like family2), and CUC1 (NAC family) (Figure 3b). In the *ERF143* promoter, the relevant CREs were enriched in the TF channels for ABI5 (bZIP family), bZIP68 (bZIP), AT4G37180 (G2‐like family1), MYB81 (MYB family), AT3G04030 (G2‐like family2), ERF043 (AP2‐EREBP), and CUC1 (NAC family) (Figure 3c). The highly relevant TF channel in the *ACS1*, *ERT2*, and *ERF143* promoters was associated with CUC1 (or NAC family) TFs that recognize nucleotide sequence motifs that are very similar to those that are bound by other NAC TFs, including NOR (O'Malley et al., 2016). This is consistent with the fact that NAC family TFs serve as a general transcriptional regulator mediating fruit ripening in various species, including tomato, peach, and kiwifruit (Gao et al., 2020; Nieuwenhuizen et al., 2021; Nuñez‐Lillo et al., 2015). However, ABI5‐like bZIP, G2‐like, and MYB81‐like TFs that bind to CREs highly relevant to the Ethyup prediction of *ACS1*, *ERT2*, and *ERF143* have not been comprehensively investigated regarding their effects on the fruit ripening process.

### ABI5‐like bZIP, G2‐like, and MYB81‐like TFs directly regulate the expression of ethylene signaling genes in kiwifruit

**Figure 4:**
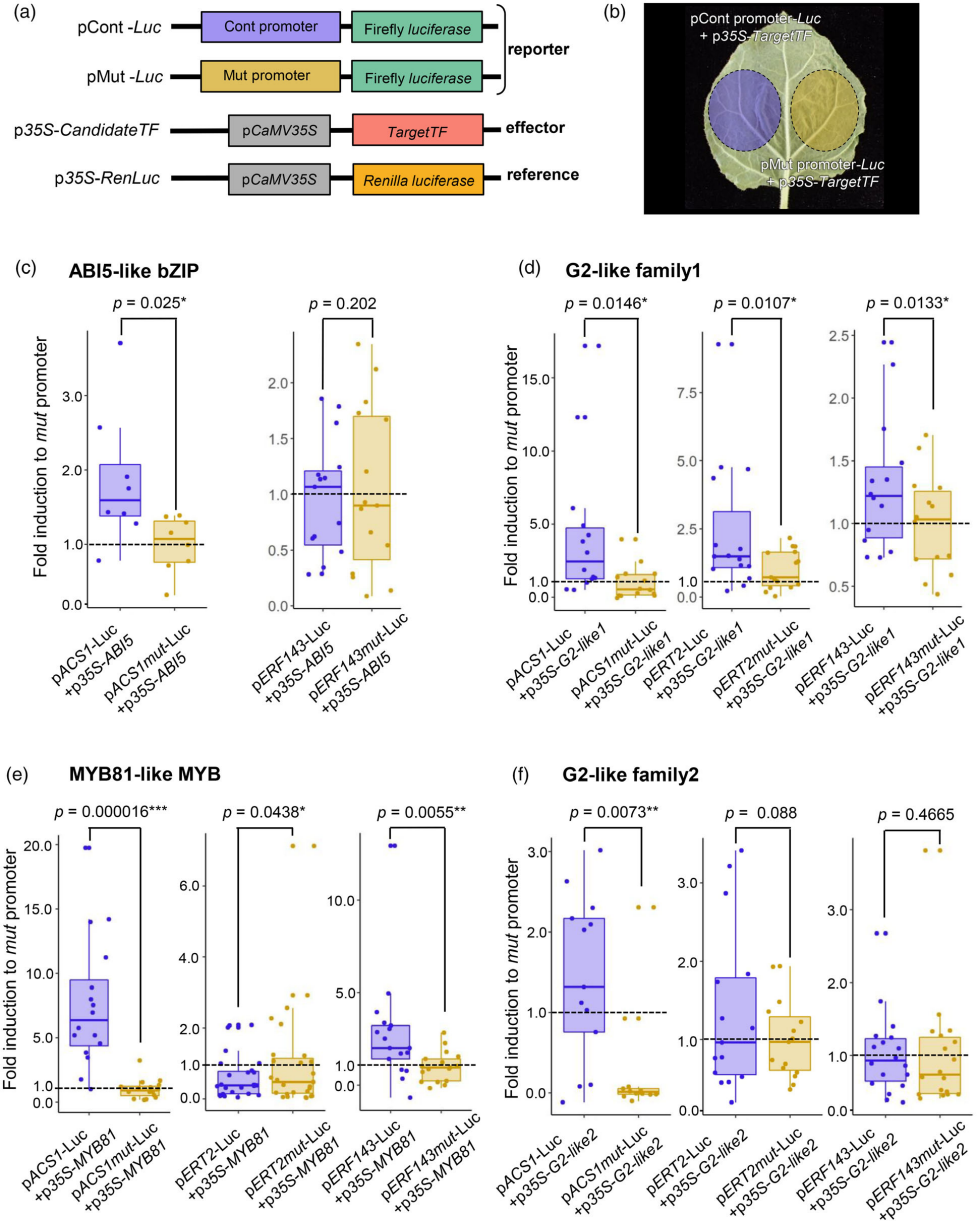
Experimental validation of the key *cis‐trans* interactions by transient reporter assay. (a) Constructs used for transient reporter assays. (b) In a leaf, both the control pCont.‐*Luc* and the mutated pMut.‐*Luc* were infected for comparison with a paired test. (c–f) Transient reporter assay with *Nicotiana benthamiana* for activation of pCont. and pMut. under the constitutive expression of candidate effector TFs (Student's *t*‐test; *P < 0.05, **P < 0.01, ***P < 0.001). (c) Examination of the ABI5‐like bZIP function as a *trans*‐factor. The pMut of *ACS1* was less activated than the pCont (*P* = 0.025) by *ABI5‐like bZIP* over‐expression. On the other hand, there was no statistical difference in activation ability between the pMut and pCont for *ERF143* (*P* = 0.202). (d) Examination of the G2‐like family1 function as a *trans*‐factor. With over‐expression of *G2‐like family1*, the pMut were statistically less activated than the pCont in all *ACS1*, *ERT2*, and *ERF143* (*P* = 0.0146, 0.0107, and 0.0133 respectively, Student's *t*‐test). (e) Examination of G2‐like family2 as a *trans*‐factor. The pMut of *ACS1* and *ERT2* were statistically less activated than the pCont (*P* = 0.0073 and 0.088 respectively) by *G2‐like family2* over‐expression, while no substantial change was observed between the pMut and pCont for *ERF143* (*P* = 0.4665). (f) Examination of MYB81 function as a *trans*‐factor, the pMut of *ACS1* and *ERF143* were less activated than the pCont (*P* = 0.000016 and 0.0055 respectively), by over‐expression of *MYB81*. *ERT2* showed more activation with pMut than with pCont (*P* = 0.0438), which was however inconsistent with our hypothesis.

Guided gradient‐weighted class activation mapping (Guided Grad‐CAM) and layer‐wise relevance propagation (LRP), which are both feature visualization methods, detected key nucleotide residues constituting the CREs highly relevant to the Ethyup prediction in the *ACS1*, *ERT2*, and *ERF143* promoter regions (Figure S3). We artificially mutated these nucleotide residues to design specific promoters, including those lacking relevant CREs (pMut; Figure S3). Transient reporter assays were conducted in *Nicotiana benthamiana* leaves using the luciferase reporter gene (*LUC*) under the control of the intact promoter (pCont) or pMut as well as effector genes for the constitutive expression of TFs. The effector genes included Ethyup kiwifruit genes homologous to ABI5‐like, two G2‐like genes (AT4G37180 and AT3G04030), and MYB81 (Figure 4a). When co‐infiltrated with *ABI5‐like* (*Acc01025.1*), *ACS1* pMut was significantly less activated than pCont (*P* = 0.025, Student's *t*‐test). For *ERF143*, there was almost no difference in the activation of pMut and pCont (*P* = 0.202, Student's *t*‐test) (Figure 4c). When co‐infiltrated with *G2‐like1* (*Acc06984.1*), pMut of *ACS1*, *ERT2*, and *ERF143* was significantly less activated than the corresponding pCont (*P* = 0.0146, 0.0107, and 0.0133, respectively, Student's *t*‐test) (Figure 4d). When co‐infiltrated with *G2‐like2* (*Acc22669.1*), pMut of *ACS1* and *ERT2* was significantly less activated than the corresponding pCont (*P* = 0.0073 and 0.088, respectively), whereas there was no significant difference in the activation of *ERF143* pMut and pCont (*P* = 0.4665) (Figure 4e). When co‐infiltrated with *MYB81* (*Acc07885.1*), pMut of *ACS1* and *ERF143* was less activated than the corresponding pCont (*P* = 0.000016 and 0.0055, respectively) (Figure 4f). Although *ERT2* pMut and pCont were differentially activated (*P* = 0.0438), pMut was more activated than pCont, which was inconsistent with our hypothesis.

**Figure 5:**
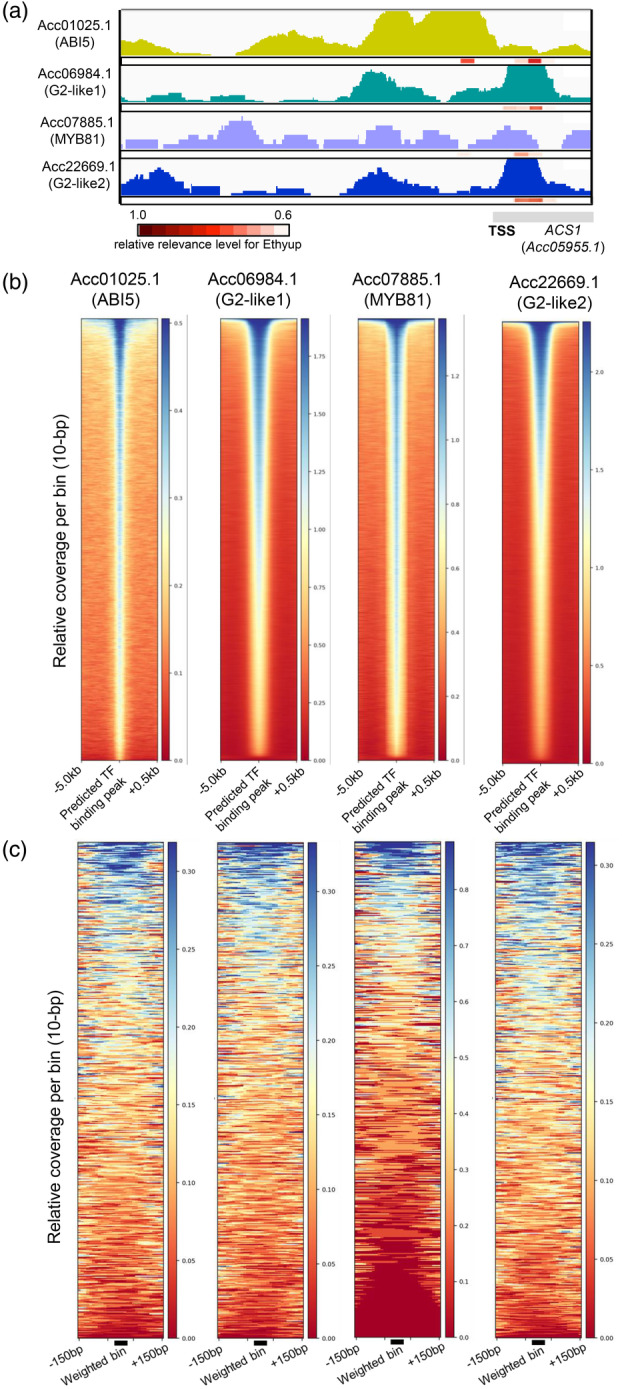
Comprehensive analysis of the binding ability of the targeted transcription factor (TF) by DAP‐seq analysis. (a) Visualization of the DAP‐seq peaks in the *ACS1* promoter region, with four TFs that can activate *ACS1* expression (Figure 4). DAP‐seq peaks overlapped the *cis*‐regulatory elements (CRE) bin relevant to Ethyup prediction in heatmaps. (b) Heatmap for the relative read coverage of DAP‐seq, with candidate TFs surrounding the predicted TF‐binding sites in the kiwifruit genome. For all four TFs, more than 90% of the DL‐predicted binding sites were covered by DAP‐seq (or TF‐binding) peaks. (c) Heatmap for the relative read coverage of DAP‐seq, surrounding the 500 highest‐confident bins relevant to Ethyup (or weighted bin). The DAP‐seq coverages tended to be high surrounding the weighted bin, but not so clear. This is firstly presumably due to that the weighted bins are not always under the DAP‐seq peak summit, as represented in panel (a). Furthermore, the numbers of the samples were much less (*N* = 500) in panel (c) than in panel (b) (*N* > 50 000), which also resulted in the visualization of coarse association between the target sites and the DAP‐seq peaks.

To examine the binding ability of these four TFs, we conducted a DNA affinity purification sequencing (DAP‐seq) analysis of kiwifruit using Halo‐TF fusion proteins and a fragmented kiwifruit genome. For instance, in the *ACS1* promoter, DAP‐seq data with ABI5‐like (Acc01025.1), G2‐like1 (Acc06984.1), G2‐like2 (Acc22669.1), and MYB81 (Acc07885.1) indicated that they formed peaks on the CRE relevant to the Ethyup prediction (Figure 5a). On a genome‐wide scale, over 90% of the CRE positions that were predicted by the DL model trained using Arabidopsis DAP‐seq data (Akagi et al., 2022) overlapped with the DAP‐seq peaks for all four TFs (Figure 5b). We also assessed the association of the X‐AI‐predicted 500 bins highly relevant to Ethyup prediction (weighted bins) and the DAP‐seq peaks (Figure 5c). Although peak signals are not so clear, DAP‐seq coverages tended to focus on the weighted bins. This ambiguous association is at least partially because of the fact that the weighted bins are not always under the summit of the DAP‐seq peak which are often broadly ranged (see Figure 5a). These overlapping/associations included 11 combinations of the four TFs and the CREs in the *ACS1*, *ERT2*, and *ERF143* promoters, which were examined in a transient reporter assay (Figure 4). Considering these results, of the 11 DL‐predicted novel key CRE–TF interactions, 7 (63.6%) directly activated expression. The lower or lack of activation associated with the other four interactions was not because the TF was unable to bind to the CRE. More likely, it was due to an inaccurate prediction of expression by the explainable CNN models or a lack of co‐factors in the reporter assay.

### Potential correlation between the explainable DL and the co‐expression network

**Figure 6:**
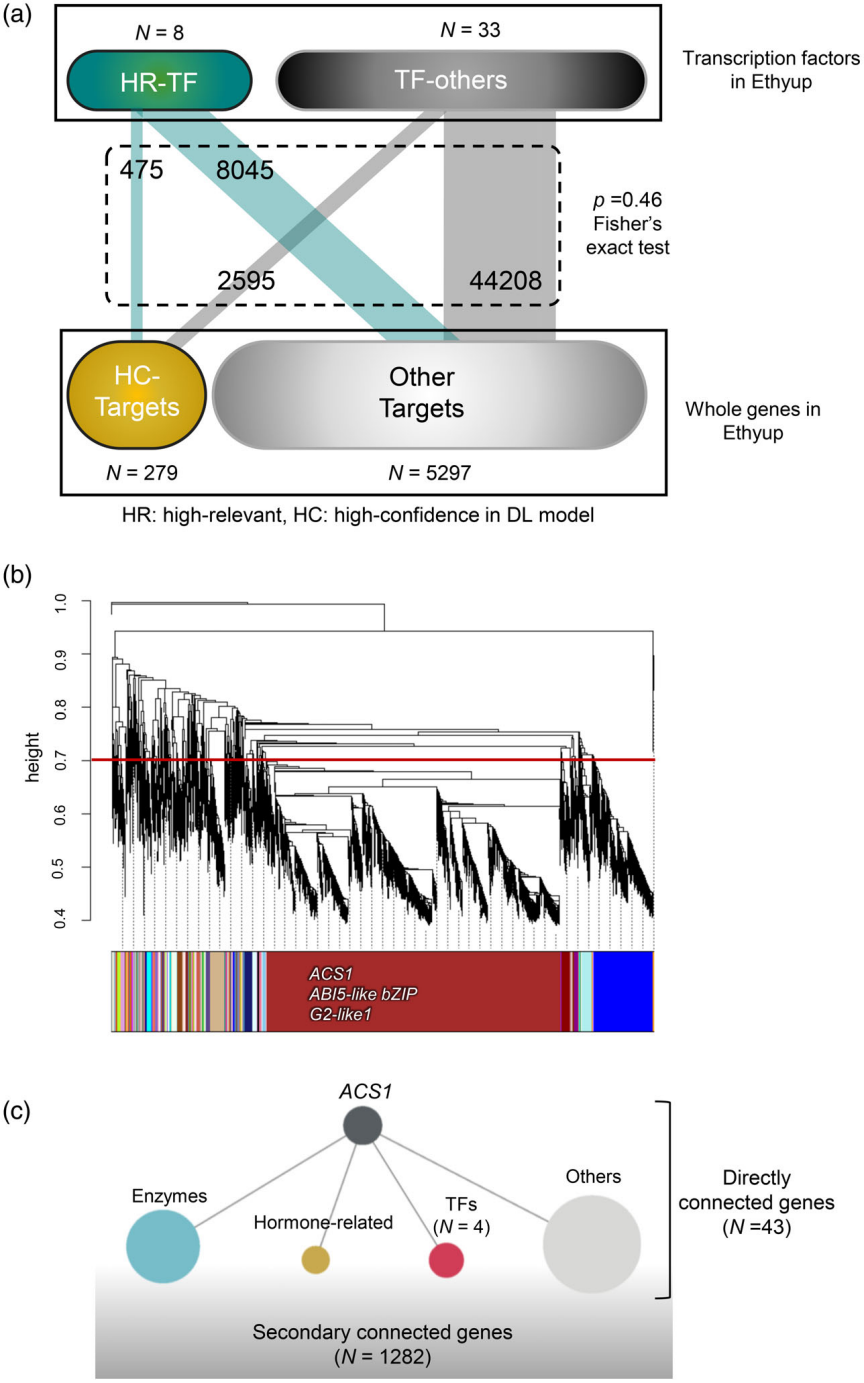
Mutual complementation in co‐expression network and explainable deep learning analyses. (a) In the first‐degree (or direct) gene connections in the weighted gene co‐expression network analysis (WGCNA) co‐expression analysis of Ethyup genes (*N* = 5576), the connections of the transcription factors (TFs) with high relevance in our convolutional neural network (CNN) model (HR‐TF, the highest 20% relevant TFs) and their high confidence target genes (HC‐target, the highest 5% confidence genes) were not significantly enriched (*P* = 0.46, Fisher's exact test). (b) In the WGCNA analysis, when clustering is performed at height = 0.7 in the dendrogram, two *cis‐trans* pairs directly proven by experimental validations, *ACS1*‐*ABI5‐like bZIP* and *ACS1*‐*G2‐like1*, are nested into the same module. (c) Genes annotations are directly and secondarily connected to *ACS1* in the module containing *ACS1*, *ABI5‐like bZIP*, and *G2‐like1*. The genes directly (*N* = 43) and secondarily (*N* = 1282) connected to *ACS1*, did not include *ABI5‐like bZIP* and *G2‐like1* genes.

A weighted gene co‐expression network analysis (WGCNA) (Zhang & Horvath, 2005) of the Ethyup genes (*N* = 5,576) detected highly correlated gene connections (weight >0.4 for the topological overlap matrix similarity). We examined the potential correlation between the first degree (or direct) connections in the co‐expression network and the CRE–TF interactions predicted by our CNN model. For *ACS1* and its predicted regulatory TFs, including the ABI5‐like TF (Figure 3a), we identified the gene pairs comprising highly relevant TF genes in our explainable DL model (top 20% relevance) and their targeted genes predicted with high confidence (top 5% confidence). Notably, these pairs were not significantly enriched among the first degree connections revealed by WGCNA (*P* = 0.46, Fisher's exact test; Figure 6a). We focused on the gene pairs (or CRE–TF interactions) confirmed by the transient reporter assay (Figure 4) and DAP‐seq analysis (Figure 5). They were often nested in the same module according to WGCNA (clustered with a height of 0.7 in the dendrogram). *ACS1*, *ABI5‐like bZIP*, and *G2‐like1* were classified in the same module (Figure 6b). This module (top 3% weight) included 43 genes that were directly connected to *ACS1* (Table S6). Some of these genes encoded expression regulators, such as zinc finger (AN1‐like) family proteins (PMZ and SAP12), as well as ethylene‐related enzymes, including those associated with ethylene biosynthesis (EFE, ACO4, and EAT1). However, *ACS1* was not connected to either *ABI5‐like bZIP* or *G2‐like1*. The genes with second degree connections to *ACS1* (*N* = 1,282) did not include *ABI5‐like bZIP* and *G2‐like1*, although they were relatively highly correlated with *ACS1* in terms of expression patterns (*r* ^2^ > 0.89). These results imply that our explainable DL model predicted the regulatory effects of TFs on gene expression at least somewhat independently of co‐expression patterns and may be applicable for identifying novel CRE–TF interactions, thereby representing an approach that differs from co‐expression network analyses. Therefore, integrating explainable DL and co‐expression network analyses, which involve two independent methods, may enable a thorough interpretation of *cis*–*trans* regulatory networks.

## DISCUSSION

In our explainable DL model for predicting Ethyup genes, nine relevant TFs (i.e., ABI5, bZIP68 [bZIP family], LBD13 [LOB‐AS2 family], G2‐like1, G2‐like2, MYB81, MYB99 [MYB family], CUC1 [NAC family], and ERF043 [ERF‐EREBP family]) may be novel regulators important for ethylene‐induced upregulated expression. Of them, the NAC and ERF‐EREBP families comprise TFs related to fruit ripening in many species. ABI5 (bZIP family) TFs reportedly contribute to stress‐induced responses (Skubacz et al., 2016). A recent study indicated these TFs are associated with cold stress‐related fruit abnormalities (Song et al., 2022). Although ABI5 affects *ACS1* expression in maturing plum fruits (Sadka et al., 2019), its relationship with fruit ripening has not been characterized. The G2‐like family mediates chlorophyll biosynthesis during fruit maturation (Nguyen et al., 2014; Powell et al., 2012), but its direct effects on fruit ripening remain unclear. There are no reports indicating that ABI5 and G2‐like are related to maturation in Arabidopsis or rice. In the MYB family, MYB70 is involved in ethylene‐triggered tomato fruit ripening (Cao et al., 2020). However, MYB70 and our targeted MYB81 (Dubos et al., 2010) belong to distantly related subfamilies. Although there are reports of MYB being related to maturation in Arabidopsis or rice (Liu et al., 2011; Zhang et al., 2017), none of them belong to the same clade as MYB81 in this study. The effects of the LOB‐AS2 family on fruit ripening have been reported, but only for bananas (Ba et al., 2014), where MaLBD may function as a transcriptional activator regulating the expression of fruit ripening‐related genes (e.g., *MaEXP1/2*). For most of the highly relevant TFs for the predicted Ethyup genes, their direct involvement in fruit ripening, particularly in tomatoes, has not been reported. Although it may be possible to think the possibility that these TFs involving fruit ripening have not been identified in tomatoes, our results suggest that key TFs related to kiwifruit ripening may have evolved in a lineage‐specific manner, similar to tomato TFs, including *RIN* (TGC, 2012). Therefore, to comprehensively identify TF–CRE interactions, it may be necessary to apply the available information for a few model crops, while also constructing interacting models independently in each lineage. Our results, which were obtained from analyses of only small sets of transcriptomic data, may be useful for identifying lineage‐specific novel TF–CRE interactions in a non‐model species.

To detect relevant TF–CRE interactions, we must consider the fact that the CNN layers merge the collinearity using a pooling function, which may result in the merging of similar CREs recognized by different TFs. For example, in this study, although we detected the ABI5‐binding CRE as a key motif for predicting Ethyup genes, we should also consider the many homologous TFs that may recognize CREs that are similar to those targeted by ABI5‐like bZIP. Indeed, the expression patterns of such homologous TFs may be exploited to identify the most likely candidate *trans*‐factors. Except when the objective expression change is due to a genetic mutation in a *trans*‐factor, upregulated gene expression depends on the upregulation (or activation) of the corresponding regulators. Furthermore, in addition to CRE information, complex regulatory grammar such as interactions between TFs, enhancers, and epigenomic information would naturally have a significant impact on gene expression patterns. However, the DL‐based approach in this study identifies regulatory factors within a scope that does not take these influences into account. Hence, consideration of further complicated input may achieve higher performance in expression prediction. The issue arises, however, that increasing the amount of (complicated) information can lead to ambiguity in the factors identified by X‐AI. On the other hand, by purposely limiting the information to simple TF binding, there may be the advantage of detecting factors that can be explained solely within that specific context.

Our explainable DL‐based approach did not produce results that were perfectly consistent with those of the co‐expression network analysis, which predicts gene connections on the basis of expression similarities (or correlations). This inconsistency may be because our explainable DL model detects direct CRE–TF interactions independently of the proportional relationship of their expression patterns. The expression patterns of some TF genes are not highly correlated with the expression patterns of the targeted genes, as exemplified by the bHLH and/or WDR TFs of the MYB–bHLH–WDR complex. This complex mainly regulates secondary metabolism‐related pathways, in which MYB TFs often have specific regulatory functions, whereas the bHLH and WDR TFs have more general functions (Feller et al., 2011; Xu et al., 2015). Hence, *cis–trans* interactive networks may not always be explainable if only the correlated expression of TF genes and the targeted genes is considered. Nevertheless, co‐expression network analyses remain highly useful. Our results indicate that multi‐level approaches that involve analyses of co‐expression data, as well as AI‐guided intuition, may provide additional novel insights into *cis–trans* interactive networks.

## EXPERIMENTAL PROCEDURES

### Construction of CRE arrays for the kiwifruit genome

We applied the fully connected DL models trained with the Arabidopsis DAP‐seq data (O'Malley et al., 2016), which predicted CREs bound by each of the 370 TFs (Akagi et al., 2022). We predicted CREs in the 2‐kb promoter sequences (5′ promoter regions 1.5‐kb upstream to 500‐bp downstream of the transcription start sites) of the 33,044 genes in the kiwifruit genome (*N* = 33,044) (Table S4). We excluded genes if their 2‐kb promoter regions overlapped with the adjacent genes. According to Akagi et al. (2022), promoter sequences were analyzed with a sliding window (31 bp bins, 2 bp steps), and binarized the prediction confidence with the threshold = 0.8 in 50‐bp bins, to make a one‐dimensional binary CRE array per gene for each TF.

### Mining of transcriptomic datasets of ripening kiwifruit

We reanalyzed a transcriptomic dataset of three replicates of kiwifruit mesocarp 5 days after treatment with 5000 ppm exogenous propylene (an ethylene analog) and the control pericarps at harvest (Asiche et al., 2018). The Burrows–Wheeler Aligner (v0.7.15) (Li & Durbin, 2009) was used to map mRNA reads to the kiwifruit reference sequences (Pilkington et al., 2018). The mapped reads were calculated as the RPKM (reads per kilobase per million mapped reads) for normalization. For genes with at least one read mapped to the gene (*N* = 27 656), differentially expressed genes between the pericarps 5 days after propylene treatment (5000 ppm) and the control pericarps at harvest were detected using DESeq (Love et al., 2014). Genes that were upregulated or downregulated in the ethylene‐treated samples (Ethyup and Ethydown genes, respectively) with FDR <0.05 and >1.5, 3, 5, 10, 20‐fold change (Table S1), were used for the explainable DL classification analyses. For the bias test, genes with RPKM values ≤1.0 were calibrated to RPKM = 1.0 to avoid exaggerated bias values.

### DL models for predicting expression patterns from CRE arrays

All the genes (*N* = 33 044) applicable to the DL test were assigned 25% for testing and 75% for training/validation sets, creating four cross‐validation sets. The training/validation sets were randomly selected with 70% for training and 30% for validating. These training/validation sets were applied to the previously constructed one‐dimensional CNN model (https://github.com/Takeshiddd/Genome‐wide‐cis‐decoding‐for‐expression‐designing‐in‐tomato‐with‐cistrome‐and‐explainable‐deep‐lear; Akagi et al., 2022). We investigated kernel size (3–20), layer depth (3–16 CNN layers), epoch numbers (5–200), learning rates (0.001–0.00001), optimizers (NAdam and SGD), and decay to optimize the model's performance in each classification task for at least 40 times. Then, we selected the hyper‐parameters that give the best performance on the validation set. In comparing the two classes, the ratio of the number of genes included in each class was used as a “class‐weight” option to correct the imbalance between the two classes. As an index of the accuracy of the prediction models, we visualized the ROC‐AUC curve by plotting the true positive‐false positive rate in the testing samples.

### GO enrichment analysis

The Arabidopsis (TAIR 10) ortholog of the Ethyup genes with the highest 5% confidence in kiwifruit (*N* = 279; Table S3) was identified using blastx (*P* < 1e‐5).

The Arabidopsis ortholog was used as the query gene for Agri GO (http://systemsbiology.cau.edu.cn/agriGOv2/) and GO terms (FDR < 0.05) in the enriched Biological Process category were identified.

### Feature visualization in DL predictions

We applied the previously constructed feature visualization codes (https://github.com/uchidalab/softmaxgradient‐lrp and https://github.com/Takeshiddd/Genome‐wide‐cis‐decoding‐for‐expression‐designing‐in‐tomato‐with‐cistrome‐and‐explainable‐deep‐lear; Akagi et al., 2022), to identify the relevance in each CNN prediction model. Briefly, we used Guided Grad‐CAM and LRP (Bach et al., 2015; Iwana et al., 2019; Montavon et al., 2019; Selvaraju et al., 2017) to identify key nucleotide residues in each CRE. We used guided backpropagation (Springenberg et al., 2015) to identify CRE bins relevant to predicting expression patterns.

### Transient reporter assay

We used pGWB35 (Nakagawa et al., 2007) as the reporter backbone for transient reporter assays. The 1‐kb 5′‐promoter region of the kiwifruit *ACS1*, *ERT2*, and *ERF143* were amplified from the genomic DNA of kiwifruit (*A. chinensis*) cv. ‘Rainbow Red’ by PCR using PrimeSTAR GXL DNA Polymerase (TaKaRa, Tokyo, Japan) (see Table S5 for the primer list). Point‐mutated promoter sequences were artificially synthesized by Eurofins Genomics (Tokyo, Japan), which were amplified with the primer sets to add adapter indexes. The amplified *ACS1*, *ERT2*, and *ERF143* promoter sequences were subcloned into pENTR/D‐TOPO vector (Thermo Fisher Scientific, Waltham, MA, USA) and finally cloned into pGWB35 using Gateway LR clonase II (Thermo Fisher Scientific) (pCont‐*Luc*, pMut‐*Luc*). To construct the effector vectors, *ABI5* (*Acc01025*), *G2‐like family1* (*Acc06984.1*), *G2‐like family‐2* (*Acc22669.1*), and *MYB81* (*Acc07885.1*) in the kiwifruit genome were amplified from the genomic DNA of cv. “Rainbow Red” by PCR with the identical condition as described above. The *Renilla* luciferase (*Ren*Luc) protein‐coding sequences were amplified from the pRL‐null vector (Promega, Madison, WI, USA). These amplicons were cloned into pPLV26 (De Rybel et al., 2011), of which the insert is under the control of the CaMV35S promoter, using the In‐Fusion HD Cloning kit (Clontech, Tokyo, Japan), to designate p*35S*‐TFs (for *ABI5*, *G2‐like1*, *G2‐like2*, and *MYB81*) as the effector and p*35S*‐*Ren*Luc as the reference, respectively. The primers used for constructing the effectors and reporter vectors were listed in Table S5.

To assess the ability of TFs to activate the Cont. and Mut. promoters of *ACS1*, *ERT2*, and ERF143, we performed dual‐luciferase system transient reporter assays with *N. benthamiana* leaves. The p*35S*‐TF, pCont‐Luc, pMut‐Luc, and p*35S*‐*Ren*Luc were electroporated into *Agrobacterium tumefaciens* strain EHA105. They were infiltrated into *N. benthamiana* leaves by needleless syringe using 16 and 20 biological replicates, respectively. *N. benthamiana* leaves were harvested 2 days after the infection and then applied to the Dual‐Luciferase© Reporter Assay System (Promega) to determine the firefly luciferase activity from pCont‐Luc and pMut‐Luc under constitutive expression of TFs.

### DAP‐seq analysis

Kiwifruit genomic DNA was extracted from cv. “Rainbow Red” leaves. Candidate TFs were amplified with the primer set (Table S5) and subcloned into pENTR‐D/TOPO, then cloned into the pIX‐Halo vector (O'Malley et al., 2016) as described previously (Akagi et al., 2022). DAP‐seq libraries were prepared as described previously (Bartlett et al., 2017; O'Malley et al., 2016), except that a NEBNext Ultra II DNA Library prep kit (NEB, Ipswich, MA, USA) and TNT SP6 High‐Yield Wheat Germ Protein Expression System (Promega) were used for DAP library preparation and recombinant TF expression, respectively.

### Co‐expression network

Weighted gene co‐expression network analysis (Zhang & Horvath, 2005) was performed for Ethyup genes (*N* = 5576). The first degree (or direct) connection between two genes in the co‐expression module was defined with weight >0.4 for TOM similarity. We examined the correlation between the CRE–TF interactions confidently predicted by the explainable DL and the gene connections based on the co‐expression patterns. In the direct gene connectivity in the co‐expression network, Fisher's exact test was performed to examine the possibility that the direct connectivity of the genes with high confidence for the Ethyup prediction and their highly relevant TFs in our explainable DL model is enriched. WGCNA clustering classified the Ethyup genes into 410 modules (with height = 0.7). For the module containing *ACS1*, *ABI5‐like bZIP*, and *G2‐like1* (including *N* = 3025 genes), we detected genes directly or secondarily connected to *the ACS1* gene to examine the possibility that the direct regulator of *ACS1*, *ABI5*, and *G2‐like1* closely connect to *ACS1* in the co‐expression network.

### ACCESSION NUMBERS

All analytical codes and scripts developed in this study have been deposited on GitHub and are publicly available at https://github.com/Takeshiddd/CisDecoding_cistrome. The DAP‐seq Illumina reads have been deposited on DDBJ‐DRA (bioproject: PRJDB15303, and sample IDs: SAMD00598207–SAMD00598210).

## AUTHOR CONTRIBUTIONS

TA conceived the study. SU and TA designed the experiments. EK and TK conducted the experiments. EK and KT analyzed the data. TA constructed and maintained the facilities. KT, SU, and TA developed the programs and analytic codes. EK and TA drafted the manuscript.

## CONFLICT OF INTEREST

The authors declare no conflict of interest.

## Supporting information


**Figure S1.** Prediction performance of Ethyup and Ethydown genes with various fold‐change thresholds. (a) MA plot for genes expressed in ethylene‐treated ripening kiwifruit for five thresholds. (b) ROC‐AUC values for five thresholds.
**Figure S2.** Correlation between the Ethyup prediction confidence and expression bias or abundance. Although the prediction confidence in binary categorization is often significantly correlated with quantitative biases or ranks (Akagi et al., 2020; Masuda et al., 2021), no statistically significant correlation was detected between the confidence for Ethyup predictions and expression bias, both in the Ethyup gene category (a) and in the whole gene category (b) (*R* ^2^ = 0.036 and 0.021, respectively). Expression abundance (or RPKM >1.0) in the Ethyup gene category was also not significantly correlated with the prediction confidence (c).
**Figure S3.** Visualization of key nucleotide residues relevant to TF binding in the weighted CREs in the *ACS1*, *ERT2*, and *ERF143* promoters. Guided gradient‐weighted class activation map (Guided Grad‐CAM) and layer‐wise relevance propagation (LRP) methods were used to visualize potential key residues of the CREs relevant to predicted Ethyup genes in the *ACS1* (a), *ERT2* (b), and *ERF143* (c) promoters.
**Table S1.** Ethyup gene category.
**Table S2.** Ethydown gene category.
**Table S3.** Ethyup prediction confidence.
**Table S4.** Genomic positions of gene promoter regions.
**Table S5.** Primers used in this study.
**Table S6.** Genes directly connected to *ACS1* according to WGCNA.
