# Supplementary material for: Identification of lineage‐specific cis–trans regulatory networks related to kiwifruit ripening initiation
Source: Plant J. 2024 Oct 27;120(5):1987–99. doi: 10.1111/tpj.17093 (PMC11629749; doi:10.1111/tpj.17093)
Supplement: Supplementary file 1 — Figure S1. Prediction performance of Ethyup and Ethydown genes with various fold‐change thresholds. (a) MA plot for genes expressed in ethylene‐treated ripening kiwifruit for five thresholds. (b) ROC‐AUC values for five thresholds. Figure S2. Correlation between the Ethyup prediction confidence and expression bias or abundance. Although the prediction confidence in binary categorization is often significantly correlated with quantitative biases or ranks (Akagi et al., 2020; Masuda et al., 2021), no statistically significant correlation was detected between the confidence for Ethyup predictions and expression bias, both in the Ethyup gene category (a) and in the whole gene category (b) (R 2 = 0.036 and 0.021, respectively). Expression abundance (or RPKM >1.0) in the Ethyup gene category was also not significantly correlated with the prediction confidence (c). Figure S3. Visualization of key nucleotide residues relevant to TF binding in the weighted CREs in the ACS1, ERT2, and ERF143 promoters. Guided gradient‐weighted class activation map (Guided Grad‐CAM) and layer‐wise relevance propagation (LRP) methods were used to visualize potential key residues of the CREs relevant to predicted Ethyup genes in the ACS1 (a), ERT2 (b), and ERF143 (c) promoters. Table S1. Ethyup gene category. Table S2. Ethydown gene category. Table S3. Ethyup prediction confidence. Table S4. Genomic positions of gene promoter regions. Table S5. Primers used in this study. Table S6. Genes directly connected to ACS1 according to WGCNA. [file TPJ-120-1987-s001.zip › tpj17093-sup-0001-Figures.docx]

**Supporting Information**

**Identification of lineage-specific *cis–trans* regulatory networks involving kiwifruit ripening initiation**

Eriko Kuwada^1*^, Kouki Takeshita^2^, Taiji Kawakatsu^3^, Seiichi Uchida^2^, Takashi Akagi^1,4,*^

1 Graduate School of Environmental and Life Science, Okayama University, Okayama 700-8530, Japan

2 Department of Advanced Information Technology, Kyushu University, Fukuoka 819-0395, Japan

3 Institute of Agrobiological Sciences, National Agriculture and Food Research Organization, Tsukuba, Ibaraki 305-8602, Japan

4 Japan Science and Technology Agency, PRESTO, Kawaguchi, Saitama 332-0012, Japan

*Corresponding author

Email: [takashia@okayama-u.ac.jp](mailto:takashia@okayama-u.ac.jp)

TEL: +81-86-251-8337

Running head: Lineage-specific fruit ripening regulation


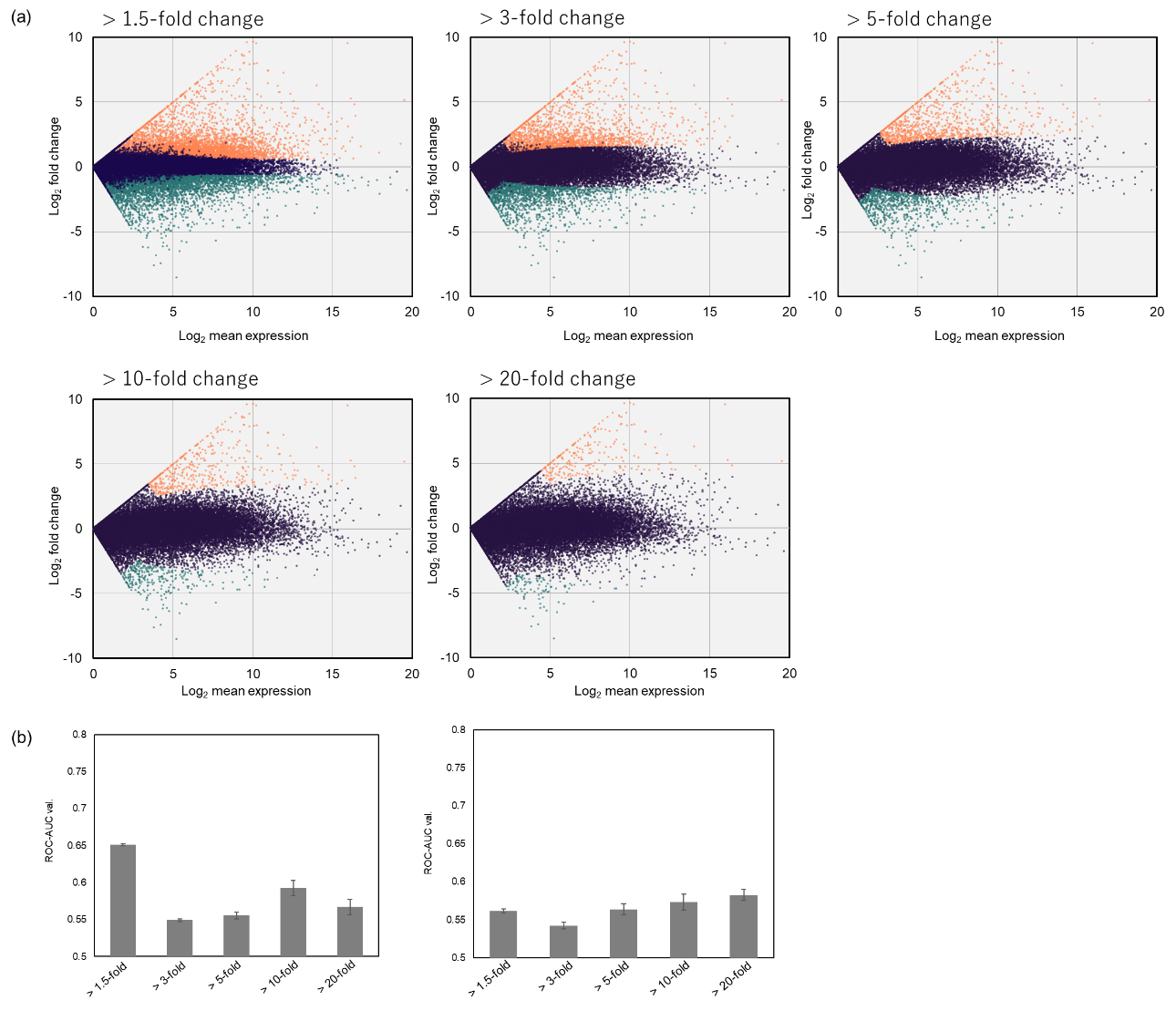


**Figure S1: Prediction performance of Ethyup and Ethydown, with various thresholds of fold-changes.**

(a) MA plot for the genes expressed in ethylene-treated ripening kiwifruit in the 5 thresholds. (b) ROC-AUC values in the 5 thresholds.


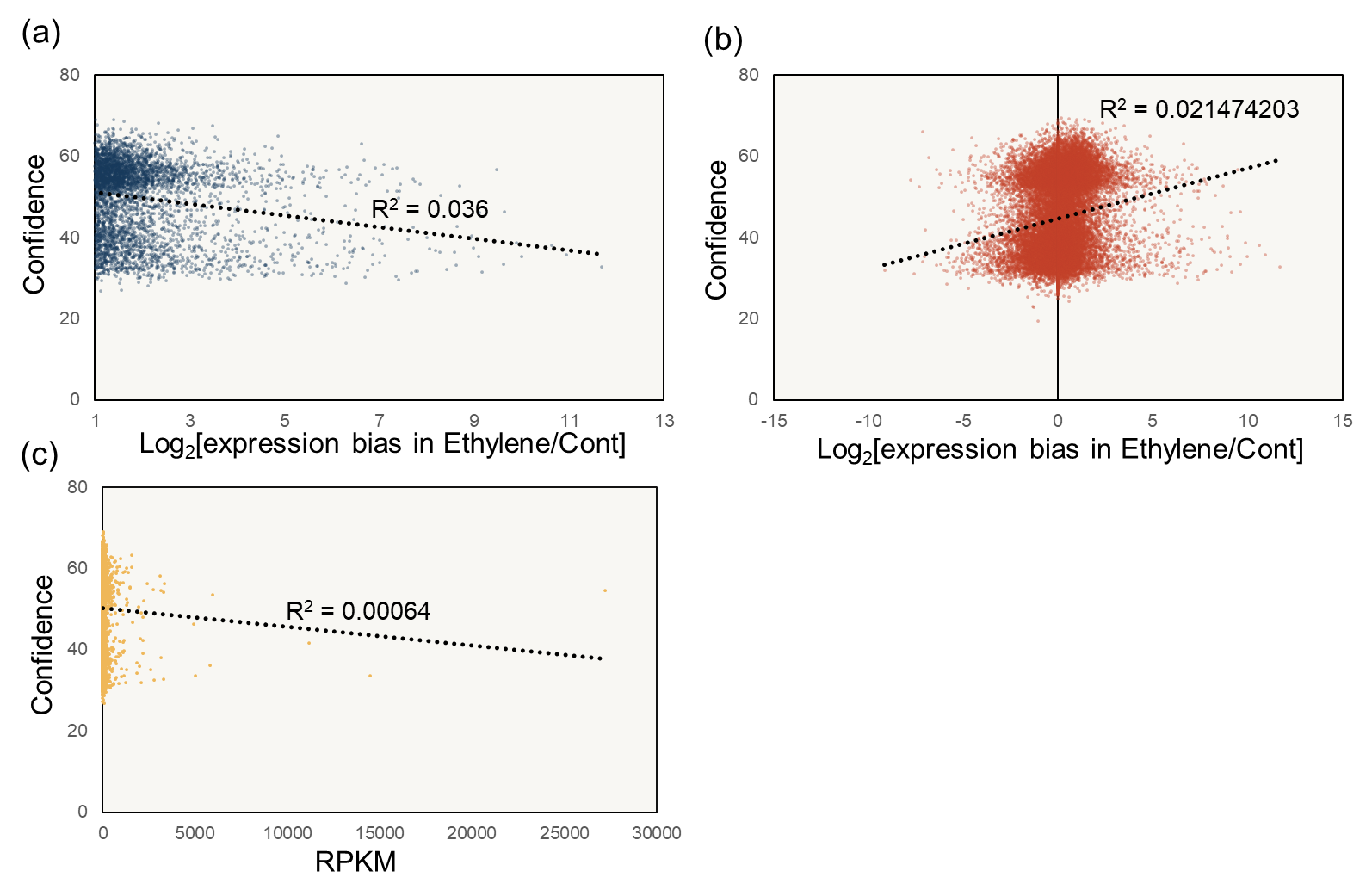
**Figure S2: Correlation in the confidence for the Ethyup prediction and the expression bias or abundances**

Although prediction confidence in binary categorization is often significantly correlated to the quantitative biases or ranks (Akagi et al., 2020; Masuda et al. 2021), no statistically significant correlation was detected between the confidence for the Ethyup prediction and the expression bias, both in the Ethyup gene category (a) and in the whole genes (b) (R^2^ = 0.036 and 0.021, respectively). Expression abundance (or RPKM, >1.0) in the Ethyup gene category also exhibited no significant correlation to the prediction confidence (c).


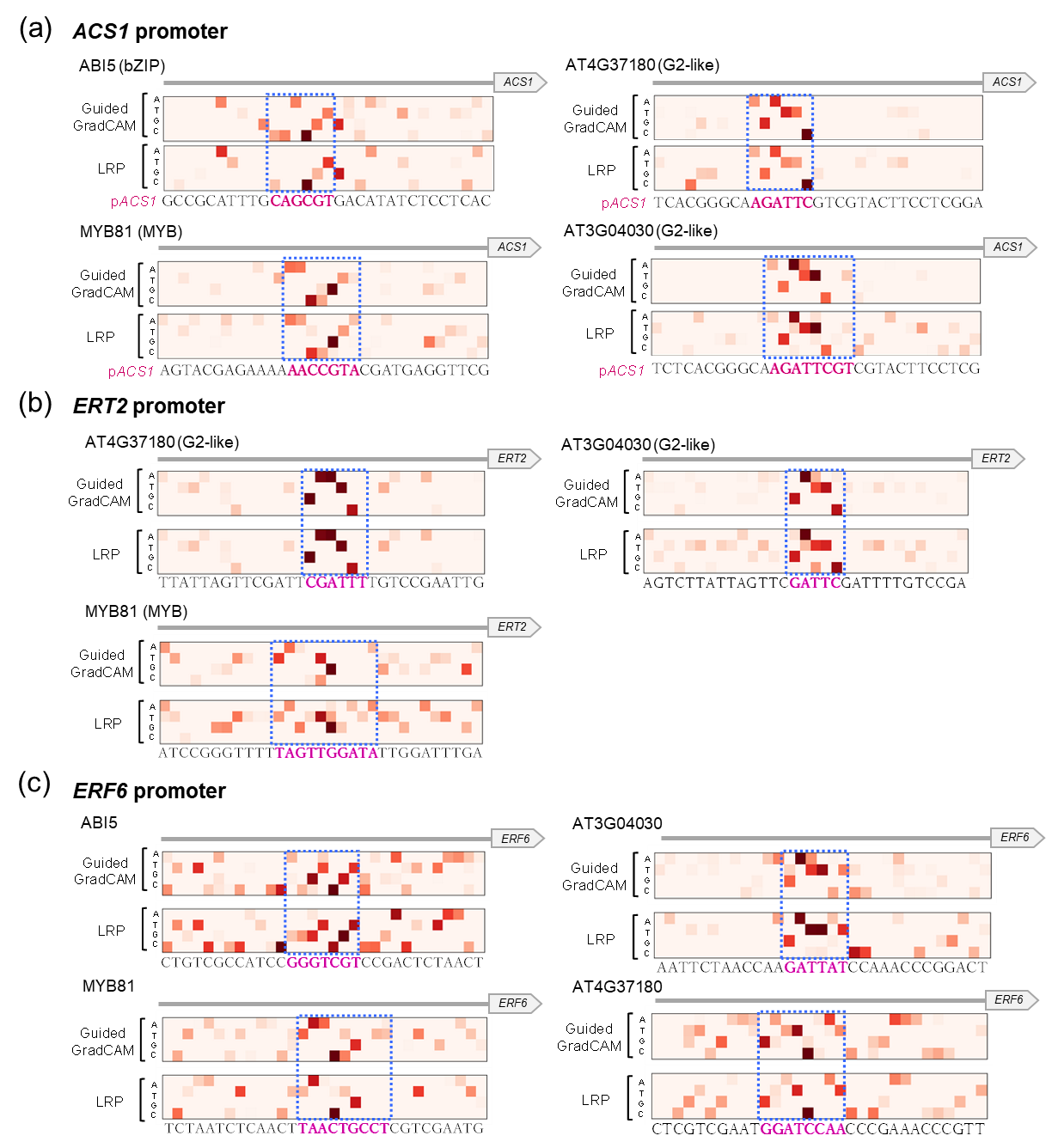
**Figure S3: Visualization of key nucleotide residues relevant to TF binding in the weighted CREs in the *ACS1*, *ERT2*, and *ERF6* promoter.**

Guided gradient-weighted class activation maps (Guided Grad-CAM) and layer-wise relevance propagation (LRP) visualized potential key residues of the CREs relevant to Ethyup prediction in the *ACS1* (a), *ERT2* (b), and *ERF6* (c) promoter.

**Table S1**: Gene list of Ethyup category.

**Table S2**: Gene list of Ethydown category.

**Table S3**: Confidences for Ethyup prediction.

**Table S4**: Genomic positions of the gene promoter regions.

**Table S5**: Primer note for this study

**Table S6:** Genes directly connected to ACS1 by WGCNA analysis

Refer to merged excel files.
